# New perspectives in the treatment of body dysmorphic disorder

**DOI:** 10.12688/f1000research.13700.1

**Published:** 2018-03-23

**Authors:** Kevin Hong, Vera Nezgovorova, Eric Hollander

**Affiliations:** 1 Department of Psychiatry and Behavioral Sciences, Autism and Obsessive-Compulsive Spectrum Program, Anxiety and Depression Program, Albert Einstein College of Medicine, Montiefiore Medical Center, The Bronx, New York, USA

**Keywords:** BDD, body dysmorphic disorder, CBT, cognitve behavioral therapy, TMS, transcranial magnetic stimulation

## Abstract

Body dysmorphic disorder (BDD) is a disabling illness with a high worldwide prevalence. Patients demonstrate a debilitating preoccupation with one or more perceived defects, often marked by poor insight or delusional convictions. Multiple studies have suggested that selective serotonin reuptake inhibitors and various cognitive behavioral therapy modalities are effective first-line treatments in decreasing BDD severity, relieving depressive symptoms, restoring insight, and increasing quality of life. Selective serotonin reuptake inhibitors have also recently been shown to be effective for relapse prevention. This review provides a comprehensive summary of the current understanding of BDD, including its clinical features, epidemiology, genetics, and current treatment modalities. Additional research is needed to fully elucidate the relationship between BDD and comorbid illnesses such as obsessive–compulsive-related disorders and depression and to develop therapies for refractory patients and those who have contraindications for pharmacological intervention.

## Introduction and context

### Clinical presentation

Body dysmorphic disorder (BDD), once called dysmorphophobia ^1^, manifests as an excessive concern with minor or wholly nonexistent defects in physical appearance; patients believe themselves to be unacceptably deformed and unattractive when actually they remain normal in appearance ^2, 3^. Patients respond to these beliefs with compulsive behaviors, such as repeatedly combing hair or covering up perceived blemishes, that are unpleasant and difficult to control ^4^. BDD is often associated with low quality of life and frequently is comorbid with major depressive disorder, substance use disorders, obsessive–compulsive disorder (OCD), and social phobias ^5– 8^. Patients often are unaware that effective treatments are available and will hide symptoms because of feelings of shame or guilt ^9, 10^. BDD is usually associated with increased suicidal ideation ^11^, delusional ideas, and poor or absent insight ^12^. The delusional variant of BDD is considered more severe ^12, 13^. Both delusional and non-delusional variants present challenges in treatment compliance; many patients seek unnecessary dermatologic, dental, and other cosmetic interventions in hopes of removing their perceived flaws ^14– 16^. These procedures typically have poor outcomes and lead to patient distress, often worsening symptoms and leading to patient dissatisfaction and loss of self-esteem ^16^. Some patients undergo repeated surgeries without achieving the expected outcome and thus have increased risk for depression and suicide.

### Epidemiology

The prevalence of BDD in the general population is approximately 2% ^17^ and is strongly associated with a history of cosmetic surgery and higher rates of suicidal ideation and suicide attempts ^17, 18^. Patients who present for cosmetic surgery treatment are also affected by BDD at rates markedly higher than in the general population, ranging from 3 to 53% ^16, 19^. Its prevalence is markedly increased in the inpatient psychiatric setting, at approximately 16% ^9^. Prevalence in outpatients with OCD, social phobia, and other disorders ranged from 10 to 40% ^9, 19^. It is often initially undetected ^10^, suggesting the importance of BDD-specific screening practices and their role in achieving better outcomes ^9, 10^.

### Pathophysiology

The pathogenesis of BDD has yet to be fully elucidated but likely involves a combination of social, psychological, and biological factors ^20, 21^. Individuals with BDD were found to be more likely to self-report a history of physical or sexual abuse (or both) in childhood or adolescence in comparison with healthy controls ^22^. A total of 78.7% of patients with BDD surveyed reported a history of childhood maltreatment, which included emotional neglect (68.0%) and emotional abuse (56.0%), physical abuse (34.7%), physical neglect (33.3%), and sexual abuse (28.0%) ^23^. These abusive experiences were also associated with a history of the following: attempted suicide, a substance use disorder, and a mood disorder. The average age of onset is about 17 years associated with gradual rather than sudden onset ^2, 24^.

An emerging body of neuroimaging research has led to the development of a working neurobiological model of BDD pathophysiology, one that involves the large-scale disorganization of neural networks involved in cognitive control and interpretation of visual and emotional information ^25^. Functional magnetic resonance imaging (fMRI) studies have shown that patients with BDD demonstrate left-sided hyperactivity in several regions within the lateral-temporal-parietal cortices upon exposure to low spatial frequency (LSF) images (which test the processing capacity of holistic information); in contrast, control subjects showed similar activity within the right hemisphere upon exposure to LSF images and showed left-sided activity only in processing of high spatial frequency (HSF) images (which test the processing of detailed featural information) ^26, 27^. This result suggests that patients with BDD will interpret even solely holistic visual information via pathways that interpret detailed, focused information. Functional network pathway analysis studies have also found that patients with BDD demonstrated hypoactive structural connectivity and poor transfer of information between primary and secondary occipital regions ^25, 28^. This may be the cause of the relative hypoactivation of the lingual gyrus, parahippocampal gyrus, and the precuneus in the aforementioned fMRI studies ^29^.

A separate fMRI study comparing visual processing in anorexia nervosa (AN) and BDD used psychophysiological interaction analyses, which estimate functional connectivity between target brain regions and the rest of the brain, to find that patients with BDD demonstrated uniquely heightened functional connectivity in occipitotemporal networks for LSF (testing the processing of holistic information) facial information ^30^. Patients with AN and those with BDD showed similar patterns of higher-order connectivity between the right fusiform face area, the precuneus, and the posterior cingulate cortex; they also showed decreased connectivity of the insula and the central opercular cortex ^25, 30^. The authors support the hypothesis that this may show impaired introspection and also cause patients with BDD and patients with AN to overattribute importance to aberrantly processed visual information ^25, 30, 31^. Another study of neural networks has also found that patients with BDD demonstrate disturbances in whole-brain structural topological organization ^32^. This study also found evidence of abnormal connectivity between regions responsible for lower-order visual processing and higher-order visual and emotional processing ^32^.

There is also evidence for a genetic component in the pathogenesis of BDD. A total of 8% of patients with BDD have a family member with similar disease, and this represents four times the prevalence in the general population ^24^. A total of 7% of patients with BDD have a first-degree relative with OCD ^33^. Although there is no clear explanation to date, this co-occurrence may be explained by genetic or environmental factors or both. A recent study in 2,148 female twins (1,074 pairs) found a covariance of genetic influences common to OCD and BDD phenotypes (64%, 95% confidence interval 0.50–0.80) ^34^. In the same study, environmental factors of influence remained unique for each phenotype. Of notice, the GABA (A)-gamma-2 1(A) allele was associated with BDD, although this did not survive correction for multiple testing ^33^. The evidence suggests that BDD is heritable and that it might share a genetic predisposition with OCD spectrum disorders, which in turn could explain their co-occurrence. More genome-wide and transcriptomic longitudinal studies are required to better understand the genetic implications of BDD and its associated molecular pathways.

### Treatment

The average age of onset is approximately 17 years and symptoms develop gradually ^2, 24^; as such, we emphasize the importance of screening in pediatric and adolescent populations, as early interventions may prevent years of risky and unnecessary cosmetic procedures ^35^. Approaches to the treatment of BDD generally consist of some combination of selective serotonin reuptake inhibitors (SSRIs) and cognitive behavioral therapy (CBT). CBT consists of a regimen of skills-focused psychotherapy centered on addressing and altering dysfunctional thought and behavior patterns with the goals of developing patient coping mechanisms and improving quality of life ^36, 37^. Therapy is focused on developing an understanding of the problems at hand and then responding via a tailored combination of behavioral experiments, exposure, and response prevention ^38^. Key strategies involve exposure with response prevention, which involves gradual confrontation of fear-inducing situations while intentionally resisting the urge to compensate via safety-seeking behaviors to reduce stress ^39^, and mindfulness interventions and perceptual retraining to help broaden focus and attend to aspects of appearance beyond self-perceived deficits ^40^. A recent meta-analysis has revealed that CBT is superior to placebo in the form of waitlist placement or other credible control treatment in the reduction of BDD symptoms with a large effect size (seven studies; d = −1.22) and is associated with improvements in insight and in delusionality with a moderate effect size (four studies; d = −0.56) ^41^. Other control conditions used include supportive therapy, psychoeducation with weekly phone calls, anxiety management, and no treatment ^41^. However, there are issues related to the use of waitlist controls. Additional CBT modules that address and focus on specific BDD-associated behaviors such as excoriation were developed and incorporated when appropriate. CBT administration via the internet is another promising modality of treatment. A recent pilot feasibility study of 23 subjects found that a pilot internet-based CBT program led to significant improvements as quantified by the Yale-Brown Obsessive–Compulsive Scale for Body Dysmorphic Disorder (BDD-YBOCS) ( *p* <0.001) with a large within-group effect size (d = 2.01) ^42^. These results suggest a need for additional, larger-scale trials of internet-based CBT, as the modality has the potential to substantially increase access to care.

Acceptance and commitment therapy (ACT) is a form of behavioral therapy based on the principle that patients consider internal processes aversive and thus can make ineffective attempts to change them ^43^. Difficulty in the interpersonal expression of emotion and experiential avoidance are suggested to be predictors of BDD symptom severity ^43^. ACT thus aims to demonstrate that avoidance is not effective and works with the patient to develop coping mechanisms and emotional acceptance. However, it has been suggested that the distinctions between ACT and CBT have yet to be clearly elucidated and that the differences may be more philosophical or theoretical than suggested thus far ^44^. A recent pilot study followed 21 subjects for 12 weeks as they received acceptance-based therapy throughout the study course. As quantified by the BDD-YBOCS, significant reductions in symptom severity before and after treatment were observed, along with a large effect size (d = 1.93) ^45^.

CBT has been established as the psychological treatment modality of choice in BDD, and a growing body of evidence shows that CBT is effective—both stand-alone and in combination with pharmacotherapy—in long-term maintenance therapy and relapse prevention ^38, 46^. A recent meta-analysis found that CBT is effective in reducing symptom severity for at least 2–4 months after treatment cessation following hourly or 90-minute sessions administered over a period of 8 to 14 weeks ^41^. SSRIs and clomipramine are favored specifically for the treatment of BDD as opposed to other serotonin and norepinephrine reuptake inhibitors (SNRIs), some of which are efficacious in conditions in which pain is the chief complaint ^47^. Serotonin reuptake inhibitors (SRIs) used for the treatment of this condition include fluoxetine ^48^, fluvoxamine ^49^, escitalopram ^50^, and clomipramine ^51^. Dosages are typically higher than those required for depression and are similar to levels seen in the treatment of OCD; in fact, dosages required often fall outside of current manufacturer recommendations ^39^. The poor insight associated with BDD also presents challenges in treatment. A combination of frequent comorbidities such as suicidality and depressive symptoms lowers the likelihood of patient adherence to treatment ^52^.

Patients should remain on medication for relatively long periods following symptom remission to reduce the likelihood of relapse ^50, 53^. A recent prospective study showed that patients who responded to a 14-week treatment of escitalopram significantly lengthened time to relapse with continued treatment for 6 months relative to placebo ^50^. Upon demonstrated reduction of BDD symptoms as quantified by the BDD-YBOCS, 18% of patients who continued treatment for 6 months relapsed, in contrast to 40% with placebo ^50^. Thus, SSRI treatment was significantly better than placebo, but a high risk of relapse remained ^50, 53^. The side effects that can be associated with SSRIs, the often-extended or even indefinite duration of treatment, a desire to discontinue medication, or the lack of access to medication are all reasons why SSRIs can be an imperfect approach to therapy. A questionnaire-based study demonstrated that depression, specifically in the forms of thwarted belonging and perceived burdensomeness, is the primary mediator of suicide risk in BDD. It is important to note the cyclical nature of these symptoms and thus the value of treating comorbid depression when treating BDD ^54^. In treatment-resistant cases, changing the SSRI used or adding a second medication may be helpful. Such augmentation medications can include second-generation antipsychotics, including olanzapine, quetiapine, and risperidone; among these, risperidone has the best demonstrated efficacy ^55^. Other options, including buspirone, administration of CBT in tandem, or switching to clomipramine, may also be beneficial ^56^. Additionally, a case study found that the addition of antipsychotics was associated with increased adherence to treatment ^57^.

A recent pilot study in adolescents developed a novel approach to CBT for adolescents that incorporated the family in exposure exercises, decreased family participation in BDD-related compulsions, and introduced mindfulness techniques and attentional/perceptual retraining ^58^. Patients who completed treatment experienced an average symptom reduction of 68% ^58^. Another case study targeted shame and anger in treatment-resistant BDD with a focused form of regular CBT ^59^. The subject of the study reported a preoccupation with facial skin associated with a history of sexual abuse and bullying ^59^. Administered CBT was enhanced with a form of imagery re-scripting, incorporation of family or support network participation, and compassion-oriented therapy ^59^. Symptom severity per the BDD-YBOCS assessment dropped by 94% throughout treatment relative to baseline, and these gains were maintained at an 18-month follow-up visit ^59^. Patients with BDD often report a history of appearance- or competency-related bullying; emotional, physical, or sexual abuse; and neglect. These patients typically retain such memories with greater detail and experience them as particularly traumatic ^22, 60, 61^. The specific goal of imagery re-scripting in this context is to restructure these memories ^61^. Imagery re-scripting involves cognitive restructuring, in which the patient explores the central negative meaning of an image and memory, and image re-scripting, which changes the nature of the relevance of the experience to the present ^61^. One case in which imagery re-scripting was used led to a 26% decrease in symptom severity, as quantified by the BDD-YBOCS, at the follow-up at 2 weeks ^61^. Another study compared imagery re-scripting with more traditional talk therapy and found that symptoms improved within the first week post-treatment, and gains were maintained for months relative to CBT alone ^62^. A key finding was that patients shifted their perception of their symptoms from the perceived physical flaw to a psychiatric condition; the discovery of insight in a condition characterized by its lack is of considerable interest ^61, 62^.

However, one issue with these techniques as proposed is the significant time and financial commitment required in adherence to a prolonged, tailored treatment with a specialist. Computerized treatment programs have been proposed and explored as an alternative modality for therapy. A computerized four-session treatment regimen that focused on interpretation bias modification was found to result in a significant increase in benign biases and reduction in threat biases post-treatment in comparison with placebo control treatment ^63^. Therapist-guided CBT provided via the internet has been found to be superior to online supportive therapy and has been found to significantly reduce symptom severity ^42^. The demonstration of a modality by which CBT can be administered via the internet has tremendous potential in increasing the access to care and in removing many significant obstacles to patient adherence ^42^.

### Classification

The *Diagnostic and Statistical Manual of Mental Disorders* (DSM) first included BDD, then called “dysmorphophobia”, in the DSM-III as an atypical somatoform disorder and did not include diagnostic criteria ^3^. It was eventually classified as a somatoform disorder in the DSM-IV, and its delusional variant was listed as a psychotic disorder ^3, 64^. In the DSM-IV, OCD was grouped in with anxiety disorders. Research since has led to a distinction between these conditions, and the DSM-V now classifies OCD and related disorders as being distinct within the category of “Obsessive–Compulsive and Related Disorders”. This new category includes BDD, excoriation disorder, hoarding disorder, and trichotillomania. Patients with BDD and patients with OCD share certain biases, which include a tendency toward perfectionism, a preference for symmetry, and repetitive compulsions ^65, 66^. There is also a basis for linking autism spectrum disorder (ASD) and BDD. The two disorders have a similar neurocognitive profile that is marked by a detail-oriented processing bias and a focus on the self ^67^. These comparisons raise the question, then, of whether treatment options have translational value. ASD and OCD have been extensively studied and characterized, and promising new treatments in these overlapping fields may provide future directions for research in BDD-specific treatment and pathophysiology.

## Discussion and future directions

The main treatments for BDD include two main first-line treatments: pharmacotherapy and CBT. However, relatively high dosages of SSRIs may lead to dose-related side effects. These symptoms are dose-related and can be attributed to their selectivity; nausea, gastrointestinal disturbances, anxiety, activation symptoms, sexual dysfunction, weight gain, and sleep disturbances figure prominently as side effects of significance ^68^. These effects are often attenuated with SSRI dose reduction ^68^. This is of importance in the management of BDD, as these side effects often present the greatest challenges to treatment adherence and BDD is a chronic disorder requiring long-term therapy. There is a need for studies that compare SSRI dosages, titration schema, and discontinuation outcomes ^56^. There is also a significant need for corresponding studies on drug efficacy and dosing in the pediatric and adolescent populations; early interventions are more likely to lead to improved outcomes, and the average age of onset is about 17 years of age. The shame and embarrassment often associated with BDD symptoms and the tumultuous nature of adolescence are likely to make such interventions difficult, and thus these feelings require focused screening techniques. Specific groups, such as children and pregnant women, may present additional challenges and raise additional questions about drug safety and efficacy. These patient populations also present clear situations in which other treatment modalities, such as CBT, may be preferred and even necessary. BDD-specific CBT techniques have been identified and studied and have been found to be effective both in reducing symptom severity and in long-term management. Preliminary studies are now available exploring the possibility of computerized delivery of therapy, which has profound implications for access to care and patient adherence.

Pharmacological and CBT-derived treatment options have proven value and efficacy in the treatment of BDD, but there is still a significant number of patients who are unresponsive to therapy. Such cases are often complex, have several comorbid conditions, and present with symptoms also pronounced in related disorders. For example, ASD and BDD may have common core symptoms targeted by novel interventions. Both of these conditions manifest with repetitive behaviors ^46^, a focus on details, and neurocognitive deficits and may well respond to SSRIs. Our group found that adults with ASD treated with fluoxetine for 12 weeks demonstrated overall improvement, especially in the domain of repetitive behaviors ^69^.

Repetitive transcranial magnetic stimulation (rTMS) involves the use of focused electromagnetic fields to stimulate specific regions within the brain; the technique is currently being studied for use in depression, psychosis, and anxiety disorders. TMS may work in part by altering the excitation/inhibition ratio ^70^. rTMS applied to the bilateral medial prefrontal cortex was found to improve symptoms in a young woman with ASD ^71^. These symptoms primarily fell into the domains of social functioning and interpersonal skills and suggest that rTMS may have broader applications in other disorders that occupy similar symptom domains, such as BDD ^71^. A recent case report found that transcranial direct current stimulation at the right temporoparietal junction in an 18-year-old patient with ASD also improved social functioning; these gains were maintained at 2 months and were also associated with a decrease in emotional lability ^72^. Bilateral deep brain stimulation at the nucleus accumbens in a 14-year-old boy with ASD led to significant improvements in self-injurious behaviors and may have promise in related contexts ^73^. A recent report on a 50-year-old woman with comorbid, treatment-resistant BDD and major depressive disorder found that electroconvulsive therapy, which has demonstrated value in the treatment of medication-resistant depression, resolved and sustained remission of both dysmorphic and depressive symptoms for 2 months ^74^. These preliminary findings, though scarce, suggest a potential role for these treatment modalities in both ASD and BDD ^69^.

Given the classification of BDD within the obsessive–compulsive spectrum disorders, there exists similar interest in novel OCD treatments. A recent retrospective study in patients with OCD suggested that low-frequency rTMS techniques have short-term utility in decreasing symptom severity; 20 sessions of low-frequency rTMS over the left orbitofrontal cortex led to a significant decrease in symptom severity relative to baseline, but no further change was reported in the following month ^75^. In another study, high-frequency deep TMS over the medial prefrontal cortex and the anterior cingulate cortex was found to reduce OCD symptom severity ^76^. Corticostriatal circuits are known to play a role in OCD and BDD, and additional targeted neurostimulation studies are needed ^77^.

## Conclusions

Research in BDD continues to evolve. Current treatment modalities are based on clinical observations, and increasingly precise strategies and standardized treatment guidelines are needed. Although traditional modalities have demonstrated efficacy and safety, there remains a significant percentage of patients who are treatment-resistant. Neurostimulation techniques, such as TMS and targeted neurosurgery, present novel directions for research informed by our understanding of the pathophysiological and neuroanatomical basis of BDD. There is also a need for readily available treatments such as computerized CBT approaches available via the internet.
